# Long term clinical outcomes of portal vein stenting for symptomatic portal vein stenosis after pancreaticoduodenectomy

**DOI:** 10.1097/MD.0000000000027264

**Published:** 2021-10-01

**Authors:** Yunghun You, Jin Seok Heo, In Woong Han, Sang Hyun Shin, Sung Wook Shin, Kwang Bo Park, Sung Ki Cho, Dongho Hyun

**Affiliations:** a Department of Surgery, Eulji University School of Medicine, 95, Dunsanseo-ro, Seo-gu, Daejeon, South Korea; b Department of Surgery, Samsung Medical Center, Sungkyunkwan University School of Medicine, 81 Irwon-ro, Gangnam-gu, Seoul, South Korea; c Department of Radiology and Center for Imaging Science, Samsung Medical Center, Sungkyunkwan University School of Medicine, 81 Irwon-ro, Gangnam-gu, Seoul, South Korea.

**Keywords:** patency, portal vein, stent, varix, venous stenosis

## Abstract

Gastrointestinal bleeding caused by portal vein (PV) stenosis is serious complication after pancreaticoduodenectomy (PD) The purpose of this study is to reveal the long-term clinical outcomes of PV stenting for symptomatic PV stenosis and risk factors of stent related complication.

Fifteen patients who underwent portal vein stenting for symptomatic PV stenosis after PD between 2000 and 2018 were retrospectively reviewed. The whole cohort was divided into 9 patients with benign stenosis group (Group-B) and 6 patients with recurrence group (Group-R).

The median follow up period was 17.0 (interquartile range 12.0–38.0) months. The technical success rate and clinical success rate was revealed at 93.3% and 86.7%. The primary patency rate of stents was 79.4% and mean patency period was 14.0 (4.0–28.0) months. There was significant difference in time to stenosis and proportion of anticoagulation treatment between 2 groups [2.0 (1.0–4.0) months vs 18.5 (2.5–50.3) months, *P* *=* .035 and 100% vs 50%, *P* *=* .044. In univariable analysis, stent diameter was found to have a significant correlation with stent occlusion (*P* *=* .036).

PV stenting was found to be feasible and safe in the treatment of symptomatic PV stenosis from a long term point of view.

## 1 Introduction

The prognosis after surgical resection for periampullary tumor is known to be dismal.^[1]^ However, advances in understanding of the tumor and adjuvant treatment after pancreaticoduodenctomy (PD) have gradually increased survival rates.^[2,3]^ Accordingly, clinical focus in long term complications occurring after PD has increased. In recent decades, several studies on portal vein (PV) stenosis, one of the long-term complications that occur after PD, have been conducted. Among these, 1 single center study reported that the incidence of PV stenosis reached 19.6% after PD.^[4]^

Tumor recurrence, postoperative change, radiation therapy, and portal vein resection are known to be the causes of PV stenosis.^[4–9]^ From a long-term perspective, PV stenosis and PV occlusion can lead serious symptoms such as gastrointestinal (GI) bleeding caused by ectopic varix associated with portal hypertension.^[4,10–15]^ When a hemorrhagic event occurs, accurate diagnosis and immediate treatment are essential.^[15]^ Since surgical treatment can pose a fatal risk to patients due to severe adhesions and collateral vessels formation from previous surgery, several tertiary institutions have recently attempted radiologic interventional treatment. Recanalization of occluded PV through stenting or PV balloon dilation has shown acceptable clinical outcomes.^[5,11,16–18]^ However, little is known about complications after PV stenting and its risk factors during long term follow-up.

From this point of view, the purpose of this study is to reveal the long-term clinical outcomes of PV stenting for symptomatic PV stenosis. In addition, risk factors of stent related complication that occurred after PV stenting were analyzed.

## 2 Methods

### 2.1 Patients

Consecutive patients who underwent PD from January 2000 to December 2018 at Samsung Medical Center were retrospectively reviewed. Among of them, 15 patients treated with portal vein stenting due to portal vein stenosis. We diagnosed PV occlusion, PV stenosis and jenunal varix using contrast-enhanced computed tomography (CT). When PV diameter decreased by 50% or more before and after surgery, it was defined as PV stenosis.^[4,19,20]^ According to the definition of our center's previous studies, PV occlusion and jejunal varix were defined as follows:

1. PV occlusion means segmental discontinuation of the PV and development of collateral channels
2. Jejunal varix means clustered, tortuous, and dilated veins in the wall of the jejunum around the bilio-enteric anastomosis site.^[17]^

The cause of PV stenosis was as follows: postoperative change without recurrence (n = 6), local recurrence (n = 6), benign stricture around portal vein resection anastomosis site (n = 2) and PV thrombosis (n = 1). The whole cohort was divided into 2 groups: 9 patients with benign stenosis group (Group-B) and 6 patients with recurrence group (Group-R). PV stenting was performed in the presence of refractory symptoms such as GI bleeding or ascites caused by portal hypertension associated with PV stenosis/occlusion. This study was approved by Institutional Review Board of Samsung Medical Center, Seoul, Republic of Korea (approval number: 2020-05-141).

### 2.2 Portal vein stenting

Prior to procedure, we considered the shape, anatomical site, and extent of PV occlusion identified in contrast-enhanced CT to determine the access route for PV stenting. After the vessels involved were identified, the optimal access route was determined among the superior mesenteric vein (SMV) or splenic vein (SPV). Then, the right or left transhepatic and/or transsplenic approach was first made using the 22-G Chiba needle (Cook Incorporated, Bloomington, IN, USA) under ultrasound guidance. Following the process of inserting a 6 Fr or 7 Fr vascular sheath into intrahepatic PV or SPV, a 0.035-inch guidewire was manipulated to penetrate the occluded segment. We then advanced 4 Fr or 5 Fr angiographic catheter (either Torcon NB Advantage catheter [Cook Incorporated] or Glidecath Angled Taper [Terumo Corporation, Tokyo, Japan]) into SMV or SPV. For accurate PV stenting, the process of measuring the length and diameter of the occluded segment and confirming the collateral formation was performed using venography. After that, preballoon dilatation was carefully performed to widen the narrowed segment of vessel. PV stenting was made to cover the occluded segment completely. At this time, a self-expandable nitinol stent (SMART control, Cordis Corporation, Miami Lakes, FL, USA) was used, and the diameter was about 1 to 2 mm larger than the maximum diameter of remaining PV or SMV on the angiography. After post-dilatation using an 8- or 10-mm diameter balloon, we embolized the path for percutaneous access with coils and/or Gelfoam (Cutanplast, Mascia Brunelli S.P.A, Milano, Italy). In our institute, anticoagulation treatment was not routinely performed. Of the 15 patients who underwent PV stenting, 12 (80.0%) patients received anticoagulation treatment. The anticoagulation regimens used after stenting are summarized in Table 2. Of the patients who had received anticoagulation treatment, only 1 patient experienced a GI bleeding. This patient had received dual antiplatelet therapy [DAPT (aspirin 100 mg PO + Clopidogrel 75 mg PO)] for 28 days immediately after stenting.

### 2.3 Follow up

Clinical variables during follow up were analyzed based on electronic medical records. Time to stenosis or occlusion was defined as the time from the date of operation to the time when PV stenosis or occlusion was first detected on follow-up CT, respectively. Time to symptom is defined as the period from the date of operation to the date of symptom onset. Time to PV stenting means the period from operation until stenting occurs. Clinical endpoints of this study were technical success, clinical success, primary patency of stent and stent related complication. We defined the technical success as reopening the portal vein flow in the venogram immediately after the stent placement. Clinical success was defined as follows: after PV stenting,

1. resolution of previous symptoms without stent-related complications
2. disappearance of jejunal varix that existed before the intervention on follow up CT.

The follow-up period was defined as the period from the date of PV stenting to the last hospital visit. We defined the period of primary stent patency from the day of PV stenting to the day of stent occlusion. Stent related complication includes stent partial thrombosis or stent occlusion. The state of the stent was confirmed by follow-up CT or ultrasound examination after the procedure, which was performed at intervals of 3 to 6 months

### 2.4 Statistical analysis

Because the sample size of this study was too small, continuous variables were expressed as median with interquartile range. To compare the clinical characteristics of Group B and Group R, Student *t* tests, the Mann–Whitney test and Chi-Squared tests were used. This statistical analysis method was also used in a univariable analysis to identify risk factors affecting the 4 clinical endpoints mentioned above. Multivariable analysis using logistic regression was performed for variables whose *P* value was less than .05 in a univariable analysis. We calculated the primary patency of PV stent using Kaplan–Meier analysis. All statistical analyses were performed by IBM SPSS Statistics ver. 23.0 (IBM Co., Armonk, NY).

## 3 Results

### 3.1 Patient characteristics

The patient characteristics were summarized in Table 1. Among the total patients, 11 were male and 4 were female, and the average age was 61.8 years. There was no patient with liver cirrhosis. 5 patients were overweight patients (25≤ body mass index <30). The type of operation consisted of pylorus-preserving PD (n = 10), pylorus resecting PD (n = 2), Whipple's operation (n = 2) and total pancreatectomy (n = 1). Preoperative bile drainage was performed in 10 patients and neoadjuvant treatment in 2 patients. In the final pathology, the most common cancer was pancreatic cancer, and it was diagnosed in 7 patients. 3 patients underwent portal vein resection. R0 resection was achieved in all 15 patients. A clinically relevant postoperative pancreatic fistula occurred in 3 patients.

**Table 1:** Patients characteristics.

	N = 15
Age (yrs)	60.0 (54.0–73.0)^∗^
Gender (male: female)	11: 4
BMI (kg/m^2^)	24.0 (23.0–26.0)^∗^
ASA classification (n, %)	
1	5 (33.3)
2	9 (60.0)
3	1 (6.7)
Pathology	
Pancreatic cancer (n, %)	7 (46.7)
Cancer of bile duct (n, %)	4 (26.7)
Ampullary cancer (n, %)	3 (20.0)
Duodenal cancer (n, %)	1 (6.6)
Operative methods (PPPD: PRPD: Whipple: Total pancreatectomy)	10: 2: 2: 1
Preoperative bile drainage (n, %)	10 (66.7)
Neoadjuvant chemotherapy (n, %)	2 (13.3)
Diameter of p-duct (mm)	3.0 (2.0–4.0)^∗^
CR-POPF (n, %)	3 (20)
Portal vein resection (n, %)	3 (20.0)
Resection margin status (n, %)	
R0	15 (100)
R1/2	0 (0)
Adjuvant treatment (n, %)	5 (33.3)
Chemotherapy only	2 (13.3)
Radiation only	0 (0)
CCRT	3 (20.0)
Symptoms (n, %)	
Hematochezia or melena	13 (86.7)
Refractory ascites	5 (33.3)
Time to symptom (mo)	19.0 (7.0–59.5)^∗^
Time to stenosis (mo)	3.0 (1.0–17.0)^∗^
Time to occlusion (mo)	8.0 (1.5–21.0)^∗^
Cause of stenosis	
Benign	9 (60)
Postoperative change without recurrence	6 (40)
benign stricture around portal vein resection with anastomosis site	2 (13.3)
PV thrombosis	1 (6.7)
Recurrence	6 (40)
Time to PV stenting (mo)	19.0 (7.0–56.0) ^∗^
Anticoagulation treatment (n, %)	12 (80.0)
Period of primary stent patency (mo)	14.0 (4.0–28.0)^∗^
Follow up period (mo)	17.0 (12.0–38.0)^∗^

ASA = American society of anesthesiologist, BMI = body mass index, CCRT = concurrent chemoradiation therapy, CR-POPF = clinically relevant postoperative pancreatic fistula, PPPD = pylorus preserving pancreaticduodenctomy, PRPD = pylorus resecting pancreaticoduodenectomy, PV = portal vein.

∗ Median (interquartile range).

The mean value of time to symptom after surgery was 19.0 (interquartile range 7.0–59.5) months. Thirteen patients experienced hematochezia or melena due to PV stenosis or occlusion. Refractory ascites developed in 5 patients. The recurrence was developed in 6 patients.

Prior to PV stenting, 3 patients received adjuvant concurrent chemoradiation therapy (CCRT) and the 2 patients received adjuvant chemotherapy. No patient received neoadjuvant radiation alone or adjuvant radiation alone.

### 3.2 Portal occlusion/stenosis

**Figure 1:**
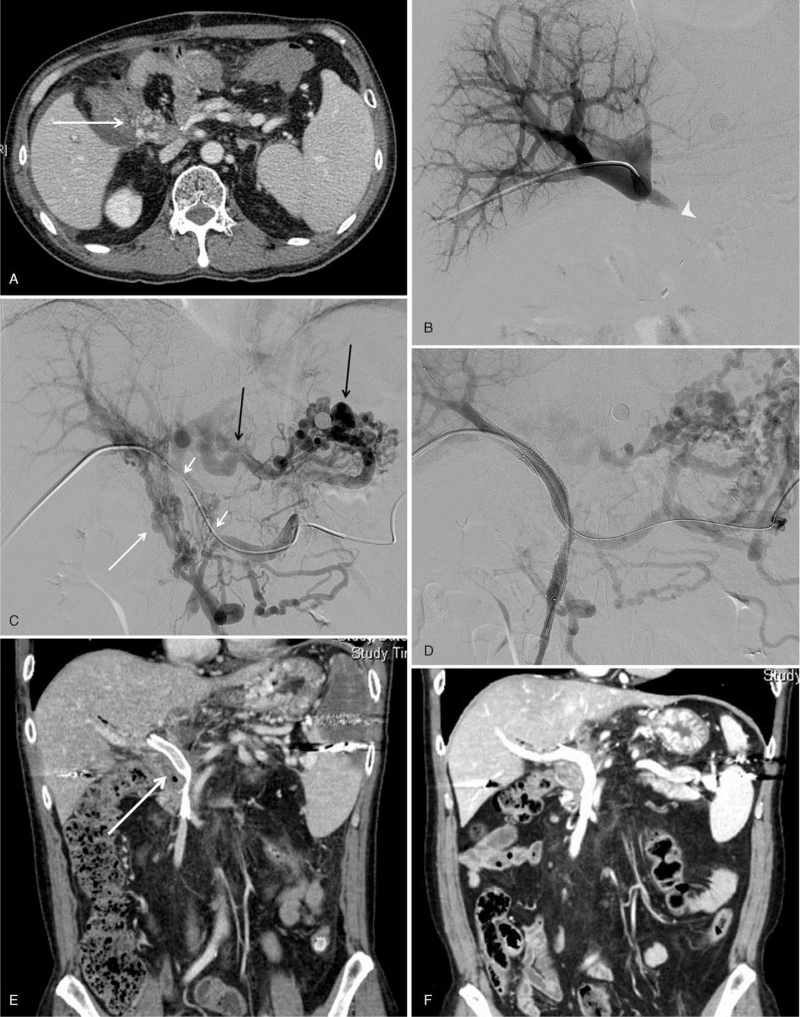
A 45-year-old male patient who underwent portal vein stenting due to recurrent hematochezia after 56 months of pylorus-preserving pancreaticoduodectomy for pancreatic cancer. (A) CT image showing varix in afferent jejunal loop (white arrow). (B) Main portal vein occlusion detected by portal venography via transhepatic approach (white arrowhead). (C) Segmental occlusion of main portal vein (short white arrows), jejunal varix connected to the right portal vein by forming a complex and extensive collateral around the afferent jejunal loop (long white arrow) and gastric varix with posterior gastric vein dilatation connected to the left portal vein (long black arrows). (D) Recanalization from SMV to MPV without jejunal collateral vessel after stenting (9 mm in diameter and 80 mm in length). (E) Improvement of varix of jejunal limb (long white arrow) confirmed on follow-up CT on the 8th day after stenting. (F) Patent stent graft shown on CT performed 78 months after stenting.

Of the 15 patients who experienced PV stenosis, PV occlusion occurred in 14 patients. Tapering ends were identified on angiography of 14 patients who experienced PV occlusion (Fig. 1). The mean follow up period was 17.0 (12.0–38.0) months. The mean value of length of lesion was 55.0 (28.0–80.0) mm. The mean value of diameter and length of stent was 9.0 (9.0 – 12.0) mm and 80.0 (60.0–80.0) mm, respectively. Except for 1 patient, 14 patients experienced PV stenosis at extrahepatic portion. The extent of involved vessel in one patient was from left main PV vein to SMV. In 12 patients, occlusion occurred to the splenoportal junction or SMV. Collateral feeding vessel to liver was observed in all patients at the time of angiography. Varix arising from the jejeunal loop formed by hepaticojejunostomy was confirmed by CT scan in 11 patients. Among these, 5 patients had esophageal varix, and 4 patients had gastric varix, which was detected by esophagogastroduodenoscopy. Percutaneous transhepatic approach was performed in 14 patients. In 1 patient, a transhepatic approach was initially attempted, but the guide wire did not pass through the occlusion site, so it was replaced with a transsplenic approach.

### 3.3 Postprocedural outcomes

**Figure 2:**
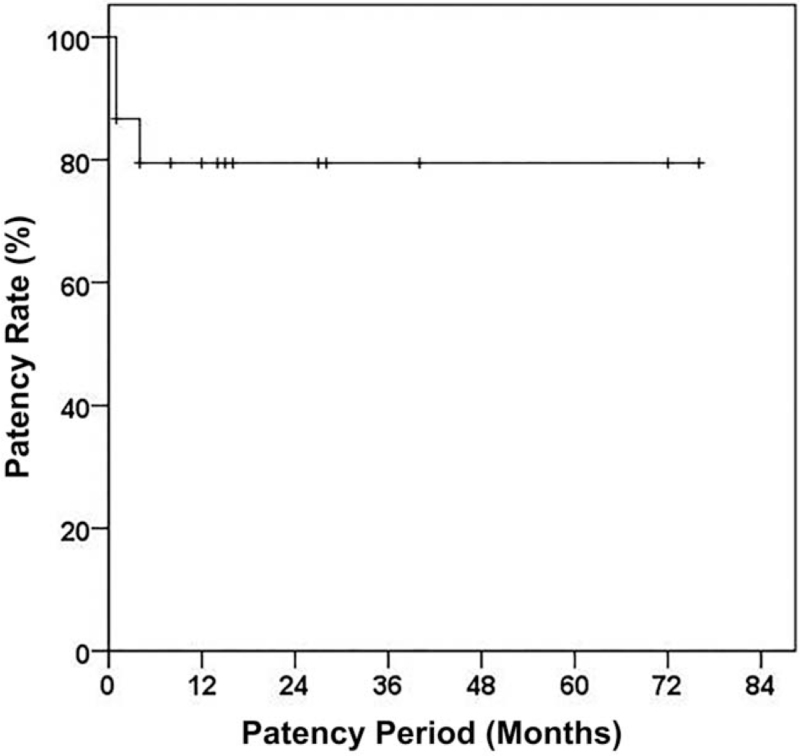
Primary patency rate of portal vein stent.

The postprocedural outcomes of 15 patients are summarized in Table 2. The technical success rate and clinical success rate was revealed at 93.3% (14 out of 15) and 86.7% (13 out of 15), respectively. The primary patency rate of stents was 79.4% and mean patency period was 14.0 (4.0–28.0) months (Fig. 2). 4 patients (26.7%) experienced stent related complication. Clinical failure occurred in 2 (13.3%) patients. One of them was 54 years old male, who underwent pylorus-preserving pancreaticoduodenectomy for distal common bile duct cancer. The R0 resection margin was confirmed and adjuvant CCRT was performed. Occlusion at main portal vein – SMV was developed 9 months after operation and GI bleeding occurred 24 month after operation. The cause of PV stenosis was tumor recurrence and the length of lesion was 70 mm. After 26 months from the day of operation, 1 stent with diameter of 9 mm and length of 80 mm was inserted into involved vessel. Technical success was achieved, and anticoagulation treatment was not performed after the intervention. However, esophageal variceal bleeding relapsed and stent occlusion caused by progression of the recurrent tumor surrounding stent occurred 36 days after PV stenting. Another patient who experienced with clinical failure was 73 years old male who received pylorus-preserving pancreaticoduodenectomy for ampulla of Vater cancer. After surgery, R0 resection was confirmed and no adjuvant CCRT was performed. Occlusions at left main PV-MPV–SMV was developed 18 days after operation and GI bleeding occurred 14month after operation. The cause of PV stenosis was fibrosis around PV associated with postoperative pancreatic fistula (POPF) and the length of lesion was 100 mm. After 16 months from the day of operation, PV stenting was performed. The involved lesion of vessel was covered by overlapping a stent with a diameter of 8 mm and a length of 60 mm and a stent with a diameter of 8 mm and a length of 80 mm. Additionally, one more stent with a diameter of 8 mm and a length of 40 mm was deployed on the distal end and balloon dilation was performed several times. In the completion venography, the stent was open, but it was still only 50% unfolded near the hepatic hilar portion, and the portal vein cavernous transformation and ectopic varix were still contrasted.

**Table 2:** Summary of 15 patients who underwent PV stenting.

Patients No. (Sex/age)	Stent graft (diameter x length)	Cause	Involved vessels	Time to stenosis (months)	Time to occlusion (months)	Time to symptom (months)	Time to stenting (months)	Technical success	Clinical success	Anticoagulation treatment after stenting	Patency	Stent related complication	Follow up period (months)
1 (M/49)	12 mm × 60 mm	Stricture around PV R&A	MPV-SMV	15	57	180	184	Yes	Yes	Yes (warfarin + enoxaparin →warfarin)	Yes	No	16
2 (M/50)	12 mm × 80 mm	Recurrence	MPV-SMV	20	23	24	30	Yes	Yes	No	Yes	No	17
3 (M/45)	9 mm × 80 mm	Postoperative change	MPV-SMV	3	11	7	56	Yes	Yes	Yes (warfarin → DAPT^∗^ → lifelong aspirin)	Yes	Yes	78
4 (M/74)	8 mm × 100 mm 10 mm × 60 mm	Recurrence	MPV-SMV	47	52	55	55	Yes	Yes	Yes (DAPT^∗^)	No	Yes	8
5 (M/72)	12 mm × 60 mm	Recurrence	MPV-SMV	60		64	65	Yes	Yes	No	Yes	No	38
6 (F/55)	10 mm × 80 mm 10 mm × 60 mm	Postoperative change	MPV-SMV	2	7	68	71	Yes	Yes	Yes (DAPT^∗^ → aspirin)	Yes	No	26
7 (M/54)	8 mm × 100 mm	Thrombosis	MPV	1	1	7	10	Yes	Yes	Yes (heparin + DAPT^∗^ → enoxaparin + warfarin → warfarin → life long aspirin)	Yes	No	84
8 (M/78)	10 mm × 80 mm	Postoperative change	MPV	1	1	3	5	Yes	Yes	Yes (DAPT^∗^)	Yes	No	6
9 (M/54)	9 mm × 80 mm	Recurrence	MPV-SMV	3	9	24	26	Yes	No	No	No	No	35
10 (F/62)	9 mm × 80 mm	Postoperative change	MPV	5	8	7	8	Yes	Yes	Yes (DAPT^∗^)	Yes	No	41
11 (M/74)	9 mm × 40 mm	Postoperative change	MPV-SMV	2	2	7	2	Yes	Yes	Yes (DAPT^∗^ → aspirin)	Yes	No	34
12 (M/54)	10 mm × 40 mm	Recurrence	MPV	17	19	19	19	Yes	Yes	Yes (ribaroxaban)	Yes	Yes	16
13 (M/73)	8 mm × 60 mm 8 mm × 80 mm 8 mm × 40 mm	Postoperative change	Lt PV-MPV-SMV	1	1	14	16	No	No	Yes (warfarin + heparin → warfarin)	No	Yes	12
14 (F/71)	8 mm × 80 mm	Recurrence	MPV-SMV	1	7	7	7	Yes	Yes	Yes (enoxaparin → rivaroxaban+aspirin → rivaroxaban)	Yes	No	12
15 (F /60)	12 mm × 60 mm	Stricture around PV R&A	MPV-SMV	1	1	1	1	Yes	Yes	Yes (enoxaparin → rivaroxaban → lifelong aspirin)	Yes	No	17

DAPT = dual antiplatelet therapy, MPV = main portal vein, PV R&A = portal vein resection and anastomosis, SMV = superior mesenteric vein.

∗ DAPT refers to the combination of aspirin and clopidogrel.

### 3.4 Comparison of clinical variables between benign stenosis group and recurrence group

Demographic and clinical features between Group-B and Group-R are summarized in Table 3. There was significant difference in time to stenosis and proportion of anticoagulation treatment between 2 groups [2.0 (1.0–4.0) months vs 18.5 (2.5–50.3) months, *P* *=* .035 and 100% vs 50%, *P* *=* .044]. Marginally significant difference was shown in time to occlusion and period of primary stent patency [4.5 (1.0–10.3) months vs 19.0 (8.0–37.5) months, *P* *=* .091 and 16.0 (6.0–56.0) months vs 6.5 (1.0–17.5) months, *P* *=* .076]. There was no significant difference in technical success rate, clinical success rate, stent related complication and patent stent rate at last follow up between 2 groups (88.9% vs 100%, *P* = 1.000, 88.9% vs 83.3%, *P* = 1.000, 22.2% vs 33.3%, *P* = 1.000 and 11.1% vs 33.3%, *P* *=* .525)

**Table 3:** Demographic and clinical features between benign stenosis group and recurrence group.

	Group-B (n = 9)	Group-R (n = 6)	*P* value
Age (yrs)	60.0 (51.5–73.5)^∗^	64.0 (53.0–72.5)^∗^	.755
Gender (male: female)	6: 3	5: 1	.604
BMI (kg/m^2^)	24.0 (22.0–25.5)^∗^	3 24.5 (17.3–26.0)^∗^	.766
ASA classification ≥2 (n, %)	6 (66.7)	4 (66.7)	1.000
Pancreatic cancer	4 (44.4)	4 (66.7)	.608
Preoperative bile drainage (n, %)	7 (77.8)	3 (50.0)	.329
Neoadjuvant chemotherapy (n, %)	1 (11.1)	1 (16.7)	1.000
Diameter of p-duct (mm)	3.0 ± 1.6	3.0 ± 1.3	.649
CR-POPF (n, %)	3 (33.3)	0 (0)	.229
Portal vein resection (n, %)	2 (22.2)	1 (16.7)	1.000
R1/2 resection margin status (n, %)	0 (0)	0 (0)	–
Adjuvant treatment (n, %)	2 (22.2)	3 (50.0)	.329
Jejunal varix (n, %)	7 (77.8)	4 (66.7)	1.000
Esophageal varix (n, %)	3 (33.3)	2 (33.3)	1.000
Gastric varix (n, %)	3 (33.3)	1 (16.7)	.604
Hematochezia or melena (n, %)	8 (88.9)	5 (83.3)	1.000
Refractory ascites (n, %)	4 (44.4)	1 (16.7)	.580
Time to symptom (months )	7.0 (4.0–54.5)^∗^	24.0 (21.5–59.5)^∗^	.140
Time to stenosis (months)	2.0 (1.0–4.0)^∗^	18.5 (2.5–50.3)^∗^	.035
Time to occlusion (months)	4.5 (1.0–10.3)^∗^	19.0 (8.0–37.5)^∗^	.091
Length of lesion (mm)	55.0 (31.5–90.0)^∗^	53.0 (18.8–85.0)^∗^	.553
Diameter of stent (mm)^†^	9.0 (9.0–11.0)^∗^	9.5 (8.0–12.0)^∗^	.903
Length of stent (mm)^‡^	80.0 (60.0–80.0)^∗^	80.0 (55.0–85.0)^∗^	.949
Number of stent used (≥2)	2 (22.2)	1 (16.7)	1.000
Time to PV stenting (months)	10.0 (3.5–63.5)^∗^	28.0 (16.0–57.5)^∗^	.409
Anticoagulation treatment (n, %)	9 (100)	3 (50.0)	.044
Technical success (n, %)	8 (88.9)	6 (100)	1.000
Clinical success (n, %)	8 (88.9)	5 (83.3)	1.000
Stent related complication (n, %)	2 (22.2)	2 (33.3)	1.000
Patent stent at last follow up (n, %)	1 (11.1)	2 (33.3)	.525
Period of primary stent patency (months)	16.0 (6.0–56.0)^∗^	6.5 (1.0–17.5)^∗^	.076
Follow up period (mo)	26.0 (14.0–59.5)^∗^	16.5 (11.0–35.8)^∗^	.442

ASA = American society of anesthesiologist, BMI = body mass index, CCRT = concurrent chemoradiation therapy, CR-POPF = clinically relevant postoperative pancreatic fistula, PPPD = pylorus preserving pancreaticduodenctomy, PRPD = pylorus resecting pancreaticoduodenectomy, PV = portal vein.

∗ Median (interquartile range).

† When using two or more stents, the diameter of the smallest stent.

‡ When using two or more stents, the length of the longest stent.

### 3.5 Analysis of risk factor for clinical endpoints

In univariable analysis, stent diameter was found to have a significant correlation with stent occlusion (the median value of stent diameter: 8.0 (8.0–9.0) mm in patient with stent occlusion vs 9.5 (8.6–12.0) mm with no stent occlusion, *P* *=* .036). The number of stents that covered the target vessel showed a marginally significant relationship with stent occlusion (the proportion of cases using ≥2 stents: 66.7% in patients with stent occlusion vs. 2.7% with no stent occlusion, *P* *=* .081). In multivariate analysis, there was no significant risk factor for stent occlusion. There was no significant risk factor for technical failure, clinical failure and stent related complication in univariable analysis.

## 4 Discussion

The final goal of interventional treatment for symptomatic PV stenosis or occlusion is to resolve clinical symptoms and prevent related complications through successful procedures. In particular, jejunal variceal bleeding can appear as a long-term result of PV stenosis or occlusion in patients undergoing PD. This is closely related to the fact that the patient anatomically has a jejunal limb adjacent to the ventral side of PV-SMV during the PD procedure. Considering these points, PV stenting for symptomatic PV stenosis or occlusion is meaningful only when it is effective in stopping jejunal variceal bleeding as well as alleviating refractory ascites by lowering portal hypertension. The current study has shown the achievement of the aforementioned final goal of PV stenting. The results of a technical success rate of 93.3% and a clinical success rate of 86.7% support that PV stenting is an effective and safe treatment for symptomatic PV stenosis or occlusion. Also, during the long- term period, stent patency was maintained in 80% of all patients. These are comparable clinical outcomes when compared to previous studies.^[17,18,21,22]^ These promising results seem to be related to the accumulation of treatment in our institution's interventional procedures in various clinical fields over the past decades. In addition to PV stenting, we were able to gain profound clinical experience through endovascular treatment for other vascular-related diseases. The standardization of PD procedure and advance in perioperative management are also considered to have led to convincing clinical results.

In the early days when interventional treatments for PV stenosis or occlusion were introduced, various complications were reported.^[11,23,24]^ However, with the development of the interventional technique, several recent studies since 2010 have reported clinical course of PV stenosis and acceptable clinical outcomes of PV stenting. In a study by Ohgi et al,^[21]^ PV stenosis was observed in 57 (12.4%) of 458 patients who underwent PD, and the median time to PV stenosis after operation was 32.5 months. Symptomatic PV stenosis or occlusion occurred in 7 patients, of which 6 patients underwent PV stenting. The technical and clinical success rates of the procedure were all 100%, and the stent patency was maintained in all patients during the follow up period. Kato et al reported a study on a total of 29 patients who underwent PV stenting.^[18]^ Of these, l5 patients underwent liver resection and 14 patients underwent pancreatic resection. The institution's technical success rate and clinical success rate were 100% and 81%, respectively. Stent patency was maintained in about 76% of patients. The mean follow-up period was 19.1 ± 24.9 months, and the mean stent patency period was 17.3 ± 21.4 months. The study by Shim et al was conducted in 22 patients with jejunal variceal bleeding who received PV stenting, of which 5 patients underwent liver resection, 16 patients underwent pancreatic resection, and 1 patient underwent hepaticojejunostomy.^[22]^ The technical success rate was 86.4% and the clinical success rate was 81.8%. Patent stent was observed in 72.7% of patients, and mean stent patency period was found to be about 8.1 months. Therefore, in addition to current study, the results of the aforementioned studies support that PV stenting is effective and safe in the treatment of symptomatic PV stenosis.

As far as we know, little is known about the risk factors for PV stent occlusion following PD. However, in patients who underwent hepatobiliary surgery, there are only a few studies that analyzed the factors affecting stent occlusion after PV stenting. In a study by Yamakado et al, consisting of a cohort of 40 patients who underwent PV stenting after hepatobiliary surgery, splanchnic vein involvement, cirrhotic patients classified as Child-Pugh class C, and obstruction of the portal vein system were found to be independent factors causing stent occlusion.^[25]^ Kato et al reported that the collateral vein was the only independent factor in a multivariate analysis of 29 patients.^[18]^ They argued that the reduction in blood flow to liver through PV caused by collateral vessels triggered a stent occlusion. Furthermore, it was suggested that this tendency becomes the basis that PV stent occlusion can be reduced by simultaneous PV stenting and collateral embolization. In current study, unlike the previously mentioned studies, the risk factors of stent occlusion after PV stenting were identified in only patients who underwent PD. Although no significant factor was found in the multivariate analysis, a smaller stent diameter was found to significantly affect stent occlusion in the univariable analysis (*P* *=* .036). In fact, stent diameter is also a risk factor for stent occlusion that can occur after stent placement for coronary artery disease or femoropopliteal occlusive disease.^[26–28]^ This phenomenon is presumed to occur through the following series of processes: a smaller diameter of stent will more likely cause thrombogenic flow abnormalities, which is likely to increase the probability of occurrence of thrombo-occlusive events. Therefore, if these evidences are used clinically, it is expected to be helpful in predicting stent patency after PV stenting according to the stent diameter used at the time of the intervention.

It is an interesting result of this study that the etiology of PV stenosis has opposite roles in time to stenosis and period of primary stent patency. Group-B tended to develop stenosis statistically significantly faster than group R group [2.0 (1.0–4.0) months vs 18.5 (2.5–50.3) months, *P* *=* .035]. On the other hand, the period of primary stent patency was longer in Group-B with marginally significance than in group-R [16.0 (6.0–56.0) months vs 6.5 (1.0–17.5) months, *P* *=* .076]. As the physical external force around the stent due to recurrence of cancer is applied more strongly over time, it is estimated that stent occlusion occurs more easily after a certain period of time in the Group-R. It is considered that future studies are needed to more accurately determine the effect on PV stenosis and PV stent patency according to the progress of recurrence.

This study has several limitations. First, there is a possibility that the design of a retrospective study for patients collected during the follow-up period close to 20 years acted as a bias in the study results. Second, postinterventional anticoagulant treatment policies that were not routinely applied to all patients may have influenced clinical outcomes after PV stenting. Third, there was a difficulty in supporting the statistical significance of risk factors due to the small number of cohorts. The clinical endpoints of this study are stent patency, stent related complications, technical failure, and clinical failure. As previously described in the “postprocedural outcomes” section, the number of occurrences of the above 4 clinical endpoints is 4 or less (3 patients experienced stent occlusion and 4 stent related complication. Technical failure occurred in 1 patient and clinical failure in 2 patients). The number of occurrences of the above events, which is too small for multivariable analysis, made it difficult to analyze risk factors and derive statistical significance. A robust statistical analysis will be possible if a multicenter study with a large sample is available in the future.

## 5 Conclusions

Portal vein stenting was found to be feasible and safe in the treatment of symptomatic PV stenosis after PD from a long term point of view. Univariable analysis revealed that stent diameter is a risk factor for stent occlusion. Careful observation of stent occlusion in patients treated with a small stent dimeter is required in follow up period.

## Acknowledgments

The authors would like to thank Hyemin Kim (data manager, Department of Surgery, Samsung Medical Center, Sungkyunkwan University School of Medicine) for help with data collection.

## Author contributions

**Conceptualization:** Yunghun You, Jin Seok Heo, In Woong Han, Sang Hyun Shin, Sung Wook Shin, Kwang Bo Park, Sung Ki Cho, Dongho Hyun.

**Data curation:** Yunghun You, Jin Seok Heo, In Woong Han, Sang Hyun Shin, Sung Wook Shin, Kwang Bo Park, Sung Ki Cho, Dongho Hyun.

**Formal analysis:** Yunghun You, Jin Seok Heo, In Woong Han, Sang Hyun Shin, Sung Wook Shin, Kwang Bo Park, Sung Ki Cho, Dongho Hyun.

**Funding acquisition:** Yunghun You, Jin Seok Heo, In Woong Han, Sang Hyun Shin, Sung Wook Shin.

**Investigation:** Yunghun You, Jin Seok Heo, In Woong Han, Sang Hyun Shin, Sung Wook Shin, Kwang Bo Park, Sung Ki Cho, Dongho Hyun.

**Methodology:** Yunghun You, Jin Seok Heo, In Woong Han, Sang Hyun Shin, Sung Wook Shin, Kwang Bo Park, Sung Ki Cho, Dongho Hyun.

**Project administration:** Yunghun You, Jin Seok Heo, In Woong Han, Sang Hyun Shin, Sung Wook Shin, Kwang Bo Park, Sung Ki Cho, Dongho Hyun.

**Resources:** Yunghun You, Jin Seok Heo, Sang Hyun Shin, Kwang Bo Park, Sung Ki Cho.

**Software:** Yunghun You, Jin Seok Heo.

**Supervision:** Yunghun You, Jin Seok Heo.

**Validation:** Yunghun You, Jin Seok Heo, In Woong Han, Sang Hyun Shin, Sung Wook Shin, Kwang Bo Park, Sung Ki Cho, Dongho Hyun.

**Visualization:** Yunghun You, Jin Seok Heo, In Woong Han, Sang Hyun Shin, Sung Wook Shin, Kwang Bo Park, Sung Ki Cho, Dongho Hyun.

**Writing – original draft:** Yunghun You.

**Writing – review & editing:** Yunghun You, Jin Seok Heo.
